# Genome‐wide association study reveals the genetic architecture of drought tolerance in maize using yield‐based resistance indices and transcriptomic evidence

**DOI:** 10.1002/tpg2.70255

**Published:** 2026-05-18

**Authors:** Haoyang Li, Xurong Hu, Pengyan Zhang, Yueying Zhao, Yinglu Song, Zheng Zhang, Huahu Bu, Jianzhong Chang

**Affiliations:** ^1^ Shanxi Institute of Organic Dryland Farming Shanxi Agricultural University Taiyuan China; ^2^ College of Agriculture Shanxi Agricultural University Taigu China; ^3^ Shanxi Key Laboratory of Crop Genetics and Molecular Improvement, College of Agronomy Shanxi Agricultural University Taiyuan China

## Abstract

Drought is a major environmental factor limiting maize (*Zea mays* L.) production worldwide. Unraveling the genetic basis of drought tolerance and pinpointing key loci and candidate genes are fundamental to the molecular breeding of maize with enhanced drought resistance. In this research, 200 maize inbred lines were evaluated under two contrasting water conditions—water‐stressed (WS) and well‐watered—across two consecutive years (2022 and 2023). Grain yield and drought resistance index (DRI) at maturity were determined, with DRI serving as a yield‐based index that provides a more comprehensive measure of drought tolerance. A genome‐wide association study based on the FarmCPU model was conducted. A total of 126 significant single nucleotide polymorphisms were detected, including 43 associated with yield under WS conditions (phenotypic variance explained [*PVE*] = 4.64%–10.67%) and 55 related to DRI (*PVE *= 1.47%–10.67%). By combining two independent RNA‐seq data collections, 43 core candidate genes were identified, 29 of which were functionally annotated. Gene Ontology enrichment and protein–protein interaction analyses demonstrated that these genes were primarily associated with biological processes related to metabolic regulation, signal transduction, and environmental stress responses. Notably, several transcription factors, including NAC35 (*GRMZM5G813651*), a MADS‐box protein (*GRMZM2G148693*), and a C3HC4‐type RING finger protein (*GRMZM2G147319*), have been previously implicated in drought response pathways. These findings provide new insights into the genetic architecture of drought tolerance in maize by using a mature‐plant, yield‐based DRI, which more directly reflects drought tolerance under field conditions.

## Core Ideas

- A total of 200 inbred lines were evaluated under different water conditions over 2 years.
- Genome‐wide association studies (GWAS) identified 126 significant single nucleotide polymorphisms (SNPs) linked to drought tolerance traits.
- Transcriptomic integration revealed 43 genes as key candidate genes for drought tolerance.
- The findings offer new insights into genetic mechanisms of drought tolerance in maize.

## Plain Language Summary

Drought is a major threat to global maize production. In the study, we aimed to identify genetic factors that help maize maintain yield under drought conditions. Using genome‐wide association analysis, we identified 126 genetic markers, which are DNA variations associated with drought‐related traits. By combining these results with gene expression data, we further identified 43 key genes that are involved in stress signaling, metabolism, and drought response pathways. Several important transcription factors known to influence drought tolerance were confirmed. These findings improve our understanding of how maize copes with dry conditions and provide useful information for breeding maize varieties with greater drought resilience.

**Abbreviations**
ARF: auxin‐responsive factor
BLUP: best linear unbiased prediction
DEG: differentially expressed gene
DRI: drought resistance index
GO: Gene Ontology
GWAS: genome‐wide association study
GY: grain yield
LD: linkage disequilibrium
PVE: phenotypic variance explained
WS: water‐stressed
WW: well‐watered

## 1 INTRODUCTION

Maize (*Zea mays* L.) is a vital crop worldwide, playing a crucial role as a primary source of animal feed, food, and industrial materials. The stability of maize yield is therefore critical for ensuring national food security and promoting sustainable agricultural development (Shiferaw et al., 2011). Among the various abiotic stress factors, drought represents the primary constraint limiting maize production. Drought stress occurring at critical developmental stages, such as young ear formation, leads to pronounced reductions in both yield and biomass. For example, even moderate drought stress caused a 61.4% reduction in biomass in maize (Orimoloye, 2022). Grain yield decreases by 1%–76%, depending on drought severity, timing, and developmental stage (Waqas et al., 2021). Developing and breeding drought‐tolerant maize germplasms and varieties represent the most economical and effective strategies to cope with drought stress. However, the complexity and multifactorial nature of drought stress present significant challenges to conventional breeding, such as prolonged selection cycles and limited efficiency (Hu & Xiong, 2014). The use of biotechnological approaches can greatly accelerate the development of maize varieties with enhanced drought tolerance, thereby improving resilience and yield. Understanding the genetic foundation of drought tolerance and identifying markers associated with drought‐related genes are crucial for advancing biotechnology‐driven breeding.

Genome‐wide association studies (GWAS) provide an effective approach to elucidate the genetic architecture underlying complex quantitative traits. In maize, GWAS has been widely used to identify loci associated with yield, agronomic traits, and stress resistance (Y. Liu et al., 2021; Sahito et al., 2024). Extensive GWAS analyses across diverse populations have uncovered multiple single nucleotide polymorphisms (SNPs) significantly correlated with drought resistance. S. Chen et al. (2023) identified 15 potential genes associated with drought tolerance in seedlings using an association panel of 379 inbred lines. Xue et al. (2013) reported 42 SNPs associated with drought response for grain yield (GY) and related secondary traits using a 350‐association panel. Ningning et al. (2023) detected 54 SNPs associated with drought tolerance by integrating GWAS and transcription analysis. GWAS have also successfully identified many drought‐related genes in maize, such as *ZmNAC080308* (N. Wang et al., 2021), *ZmcPGM2* and *ZmFAB1A* (Wu et al., 2021), *ZmEXPA4* (B. Liu et al., 2021), and *ZmVPP1* (X. Wang et al., 2016). Although significant progress has been achieved in identifying genomic regions associated with drought tolerance in maize, most previous GWAS have focused on seedling‐stage traits or indirect indicators, such as anthesis‐silking interval, yield components, chlorophyll content, and root‐related traits, rather than drought performance at maturity under field conditions. As a result, the genetic basis of drought tolerance evaluated at the final yield level remains insufficiently understood. To further investigate this issue, we conducted a GWAS using a mature‐plant drought resistance index (DRI) derived from grain yield under well‐watered (WW) and water‐stressed (WS) conditions. This approach was designed to more directly capture drought tolerance as reflected by final yield performance and to provide new insights into the genetic architecture of drought responses in maize.

## 2 MATERIALS AND METHODS

### 2.1 Plant materials and experimental design

A total of 200 maize inbred lines were assembled as an association panel, the majority of which originated from domestic breeding programs, supplemented by a small subset of US germplasms (Wen et al., 2025). All inbred lines were evaluated under WW and WS treatments in 2022 and 2023 at two sites, specifically Jinzhong (37.6878°N, 112.7333°E) and Xinzhou (38.4167°N, 112.7333°E) in Shanxi. The field experiment was conducted in a split‐plot design with two replicates for each treatment in each site‐year environment. Across all sites, inbred lines from both treatments were cultivated in two rows in plots (5 m long, 0.5 m apart) with a plant density of 90,000 plants/ha. WW treatment maintained adequate soil moisture throughout the whole growing season. WS treatment was applied by suspending watering during the V13 to VT stages. At other growth phases, the watering amount was half of that applied in the WW treatment. This water regime imposed drought stress during the critical reproductive transition period while allowing plants to reach maturity for yield evaluation. Other field operations were carried out in accordance with local farming practices.

### 2.2 Phenotype data collection and analyses

After maturity, effective plant numbers in each plot were recorded, and the corresponding ears were harvested. Moisture levels in the grains were determined with a PM‐8188 grain moisture tester (Kett Electric Laboratory), and the GY (kg/hm^2^) was corrected to a 14% moisture content. The yield calculation formula is as follows (Ningning et al., 2023):

$$\mathrm{GY}=\frac{W_{\mathrm{dry}}\;\times \;N\;\times \;666.67\;\times \;(1\;-\;\mathrm{GM})}{A\;\times \;(1\;-\;14\%)},$$

where GY denotes grain yield (kg/hm^2^), *W*_dry_ represents the dry weight of kernel per ear (kg), *N* refers to effective plant number, *A* stands for the two‐row plot area (m^2^), and GM indicates the measured grain moisture content.

Broad‐sense heritability and the best linear unbiased predictions (BLUP) for GY were estimated employing the *lme4* package in the R software (Xiao et al., 2016). The DRI for the respective year was determined based on BLUP values under WW and WS conditions and was designated as 2022DRI and 2023DRI, respectively. The DRI was calculated according to the following equation (Y. Li et al., 2004):

$$\mathrm{DRI}=\frac{\mathrm{GYS}.T\;/\;\mathrm{GYS}.W}{\mathrm{GYM}.T\;/\;\mathrm{GYM}.W},$$

where GYS.W and GYS.T represent the GY for WW and WS conditions, respectively, and GYM.W and GYM.T indicate the mean GY of all inbred lines for WS and WW conditions, respectively.

The broad‐sense heritability (*H* ^2^) was estimated according to the following method (L. Yang et al., 2020):

$$\;H^{2}=\frac{\sigma_{g}^{2}}{\sigma_{g}^{2}+\frac{\sigma_{e}^{2}}{n}}$$

where $\sigma_{g}^{2}$ denotes the genetic variance, $\sigma_{e}^{2}$ is the residual variance, and *n* indicates the count of sites.

**Core Ideas**

- A total of 200 inbred lines were evaluated under different water conditions over 2 years.
- Genome‐wide association studies (GWAS) identified 126 significant single nucleotide polymorphisms (SNPs) linked to drought tolerance traits.
- Transcriptomic integration revealed 43 genes as key candidate genes for drought tolerance.
- The findings offer new insights into genetic mechanisms of drought tolerance in maize.

### 2.3 Genotyping and quality control

At the V5 stage, approximately 0.1 g of young leaf was collected, quickly frozen in liquid nitrogen, and preserved at −80 C. Genomic DNA was isolated using a modified cetyltrimethylammonium bromide method (G. Liu et al., 2010). DNA purity and integrity were assessed by 0.8% agarose gel electrophoresis, and DNA concentration was determined using a NanoDrop 2000 spectrophotometer (Thermo Fisher Scientific) (Xie et al., 2019). All DNA samples were submitted to China Golden Marker Biotechnology Co., Ltd for genotyping using a 48K SNP chip. Raw SNPs were refined using the PLINK software according to a missing rate of ≤0.2 and a minor allele frequency of ≥0.05. The resulting filtered SNP dataset was used for subsequent analyses. The PopLDdecay software was used to assess linkage disequilibrium (LD) between SNP loci (C. Zhang et al., 2019).

### 2.4 Genome‐wide association analysis

The GWAS of GY and DRI was performed using the GAPIT software (V3.0) based on the FarmCPU model (J. Wang & Zhang, 2021). Population structure was adjusted using the first three principal components. To evaluate model fitting and the control of population structure, the genomic inflation factor (*λ*) was calculated based on the median of the observed chi‐square statistics divided by the expected median of the chi‐square distribution with one degree of freedom (0.455) (Tsepilov et al., 2013). The R package CMplot was employed to generate Manhattan plots. Considering the exploratory nature of this study, a significance threshold of *p *< 1×10^−3^ was used to identify suggestive association loci, following previous plant GWAS studies (J. Chen et al., 2016; L. Yang et al., 2020). This threshold was selected to balance detection power and false‐positive control.

### 2.5 Functional annotation of candidate genes

Candidate genes were identified based on the LD decay interval around significant SNPs, using the B73 RefGen_v3 genome in MaizeGDB (https://maizegdb.org/gbrowse/maize_v3) as the reference. The average LD decay distance in the association panel was estimated to be 200 kb; therefore, genes located within 200 kb upstream and downstream of each significant SNP were considered candidate genes (An et al., 2020). Two RNA‐seq datasets (GSE71723 and GSE132113) retrieved from the NCBI Gene Expression Omnibus were combined, including samples from tassel, ear, and leaf at four growth stages, as well as three tissues (leaf, ear, and kernel) under water stress. Differentially expressed genes (DEGs) were identified using a threshold of |log_2_fold change|≥2 to detect genes differentially expressed between drought and normal conditions. The shared DEGs between the two datasets were defined as core candidate genes. Gene clustering was performed using the *pheatmap* package in the R software (www.r‐project.org). The DAVID database (https://david.ncifcrf.gov/tools.jsp) was used to perform the Gene Ontology (GO) analysis. Protein–protein interaction was constructed using the STRING database (v12.0; https://cn.sting‐db.org/) and subsequently visualized with the Cytoscape software (Doncheva et al., 2019).

## 3 RESULTS

### 3.1 Phenotypic variation evaluation

**FIGURE 1:**
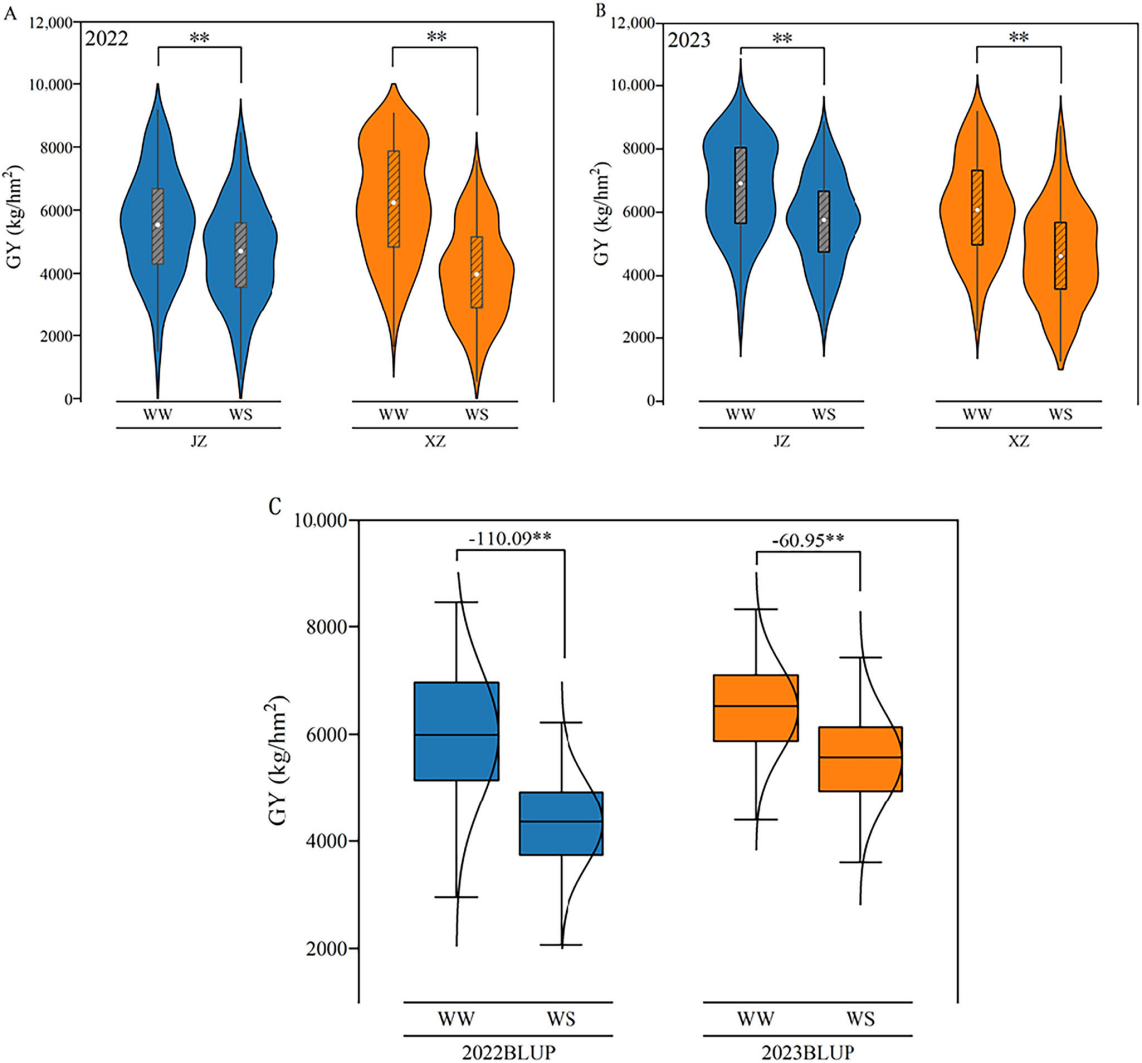
Comparison of grain yield (GY) under different locations and water conditions in 2022 and 2023. (A) GY performance in 2022; (B) GY performance in 2023; (C) GY best linear unbiased predictions (BLUPs) of two water conditions compared between 2022 and 2023. Asterisks (**) denote a significant difference at the 0.01 level. JZ, Jinzhong; WS, water‐stressed; WW, well‐watered; XZ, Xinzhou.

GY was evaluated across 2 years, two locations, and under two water regimes. In 2022, the average BLUP value under WW condition was 5988.15 kg/hm^2^, while under the WS condition, it decreased to 4336.80 kg/hm^2^, representing a 27.58% reduction. In 2023, the average BLUP values under WW and WS conditions were 6462.75 and 5548.50 kg/hm^2^, respectively, with a yield reduction of 14.15% caused by drought stress (Figure 1). Analysis of variance revealed that in 2022, except for the location effect, genotype, treatment, and their interactions (genotype × location and location × treatment), all had highly significant effects (*p *< 0.001), and genotype and environmental factors (location + treatment) accounted for 56.09% and 17.74% of overall variance, respectively. In 2023, significant effects (*p* < 0.001) were observed for location, genotype, treatment, and their interactions (genotype × location and location × treatment). Genotypic and environmental factors contributed 52.33% and 20.50% of the overall variance, respectively (Table S1). The results demonstrated that genotype was the dominant factor for yield variance, followed by environmental effects. *H* ^2^ of GY was 62% in 2022GYWS and 63% in 2022WW, with higher values observed under WW than WS conditions (Table 1).

**TABLE 1:** Summary statistics and broad‐sense heritability (*H* ^2^) estimates for grain yield (GY)

Treatment	Location	Range	Mean ± SD	CV	*H* ^2^
2022GYWW	BLUP	2377.65–8456.70	5988.15 ± 1211.7	0.20	0.63
	JZ	450.15–9172.95	5530.50 ± 1700.97	0.31	
	XZ	1649.70–9072.45	6223.65 ± 1771.95	0.28	
2022GYWS	BLUP	2070.45–6217.05	4336.80 ± 808.20	0.19	0.62
	JZ	614.4–8446.35	4633.95 ± 1606.05	0.35	
	XZ	534.75–3966.9	4012.05 ± 1490.70	0.37	
2022DRI		0.35–1.11	0.73 ± 0.15	0.21	
2023GYWW	BLUP	4397.4–8322.75	6462.75 ± 806.40	0.12	0.58
	JZ	2290.05–9985.95	6779.70 ± 1531.65	0.23	
	XZ	2229.75–9187.95	6145.65 ± 1553.40	0.26	
2023GYWS	BLUP	3603.45–7428.45	5548.50 ± 839.10	0.15	0.57
	JZ	2220.75–8850.6	5710.05 ± 1404.60	0.25	
	XZ	1270.5–8721.15	4656.60 ± 1543.65	0.33	
2023DRI		0.48–1.29	0.86 ± 0.17	0.19	

Abbreviations: BLUP, best linear unbiased prediction; DRI, drought resistance index; JZ, Jinzhong; WS, water‐stressed; WW, well‐watered; XZ, Xinzhou; CV, coefficient of variation.

### 3.2 GWAS for DRI and GY under WS conditions

**FIGURE 2:**
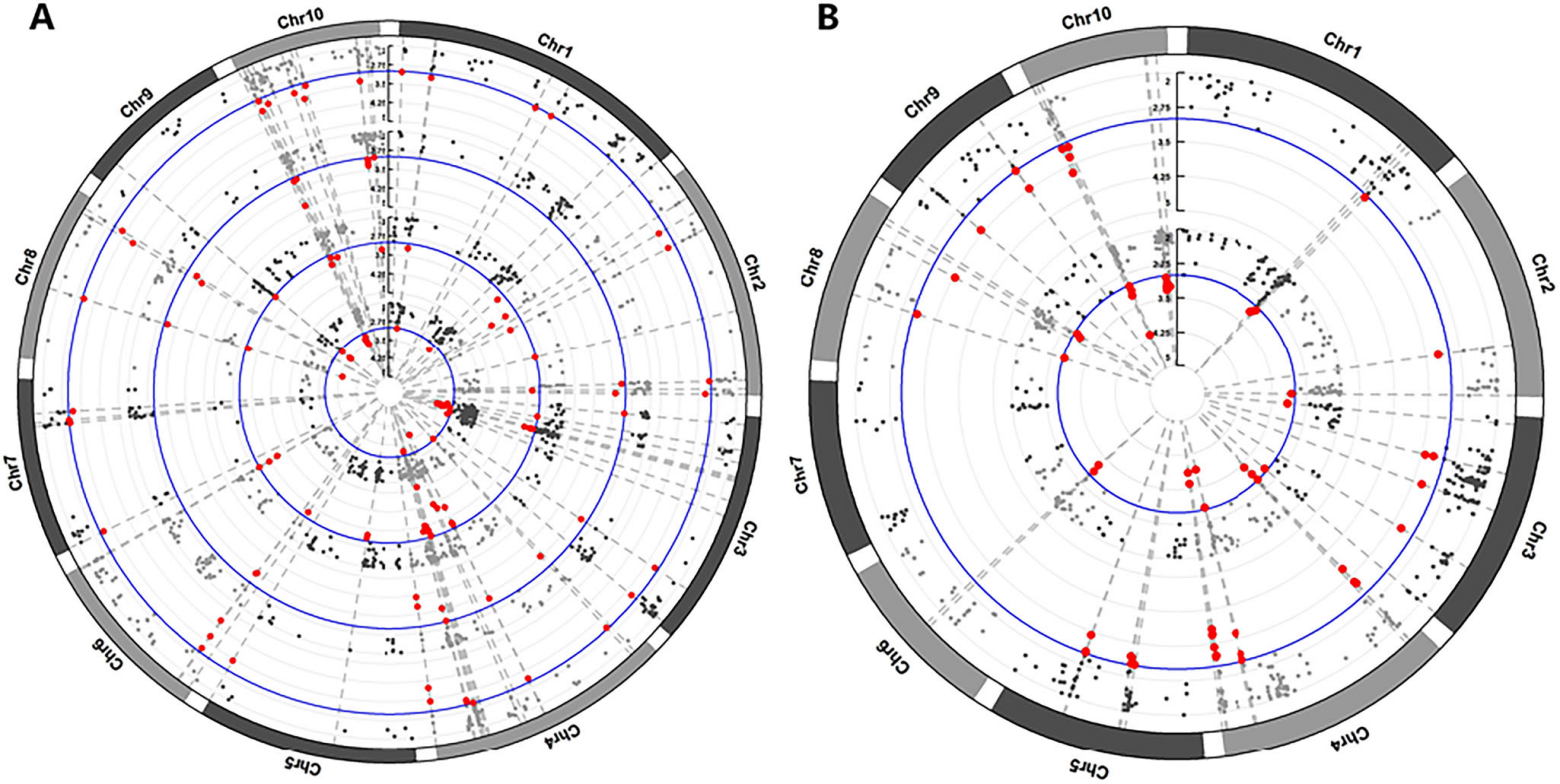
Results of genome‐wide association analysis for grain yield (GY) and drought resistance index (DRI). The blue lines represent the genome‐wide association threshold (1×10^−3^), while the red dots indicate the significantly associated single nucleotide polymorphisms (SNPs). (A) Significantly associated SNPs for GY under different water treatments in 2022 and 2023. The four concentric circles, from outermost to innermost, represent 2022GYWW, 2022GYWS, 2023GYWW, and 2023GYWS. (B) Significantly associated SNPs for DRI. The two circles from outside to inside represent 2022DRI and 2023DRI. Chr, chromosome.

GWAS was conducted using the FarmCPU model based on BLUP values for yield and DRI across two environments, with the first three principal components included to control for population structure. The genomic inflation factor (*λ*) ranged from 0.97 to 1.04, indicating effective control of false‐positive associations. In total, 126 SNPs showed significant associations (Figure 2). Among them, 55 loci were linked to DRI, with phenotypic variance explained (PVE) ranging from 1.47% to 10.67%, while 43 loci were associated with yield under WS condition, with PVE ranging from 4.64% to 10.67%. Six SNPs (Affx‐291407911, Affx‐159118475, Affx‐291441904, Affx‐291389821, Affx‐88998430, and Affx‐291406993) showed significant associations with both 2022DRI and 2023DRI and mapped to chromosomes 1, 4, 8, and 10, with PVE of 7.04%, 4.20%, 8.88%, 7.96%, 10.67%, and 8.03%, respectively. Three SNPs (Affx‐88986517, Affx‐291389821, and Affx‐88998430) were significantly associated with both 2022GYWS and 2023GYWS, located on chromosomes 4, 8, and 10, explaining 6.96%, 7.96%, and 10.67% of the phenotypic variation, respectively. Two loci, Affx‐291389821 and Affx‐88998430, showed significant associations with the traits 2022DRI, 2023DRI, 2022/2023GYWS, and 2022/2023GYWW and were positioned on chromosomes 8 and 10 (Table S2).

### 3.3 Allelic variation effects of DRI‐associated loci

**FIGURE 3:**
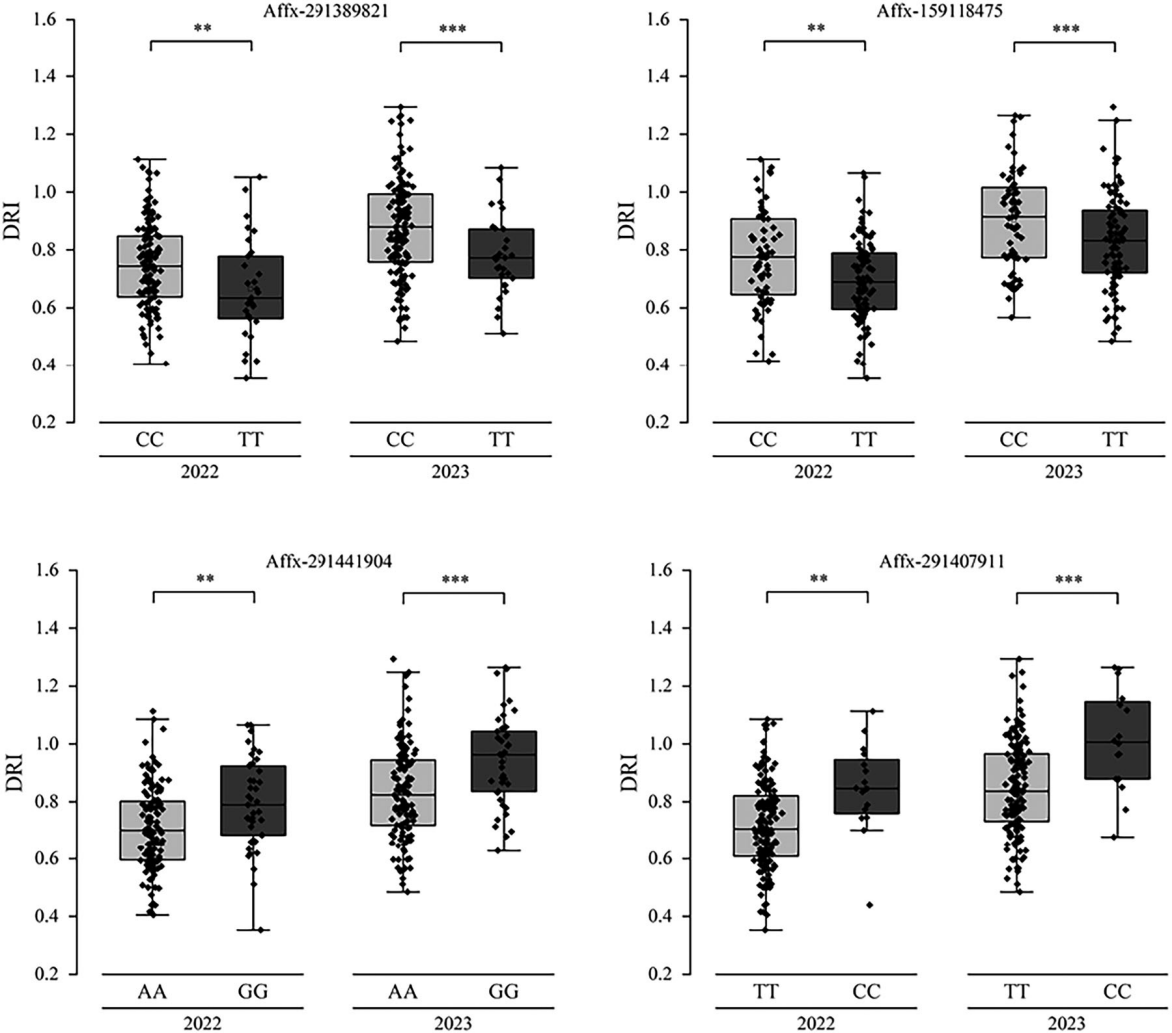
Allelic effect analysis of single nucleotide polymorphisms (SNPs) linked to drought resistance index (DRI). Asterisks (** and ***) represent significance at *p *< 0.01, and *p *< 0.001, respectively.

Six SNP markers consistently identified with DRI across 2 years, Affx‐291389821, Affx‐159118475, Affx‐291441904, and Affx‐291407911, were selected for allelic effect analysis (Figure 3). Affx‐291389821 (T/C) divided the inbred lines into T and C genotypes, with C identified as the favorable allele. Inbred lines carrying the C allele showed significantly higher DRI than those with T allele, with increases of 0.08 and 0.10 in 2022 and 2023, respectively (*p* < 0.01). Affx‐159118475, also a T/C variant, showed a similar trend: the C allele was associated with a 0.07 increase in DRI in both years compared to the T allele (*p* < 0.01). For Affx‐291441904 (G/A), genotypes with G allele showed greater DRI values than A‐allele genotype, increasing by 0.08 in 2022 (*p *< 0.01) and 0.12 in 2023 (*p* < 0.001). At Affx‐291407911 (T/C), the presence of the C allele conferred an advantage, with C‐type lines showing DRI increases of 0.13(*p* < 0.01) and 0.16(*p* < 0.001) over T‐type lines in 2022 and 2023, respectively.

**FIGURE 4:**
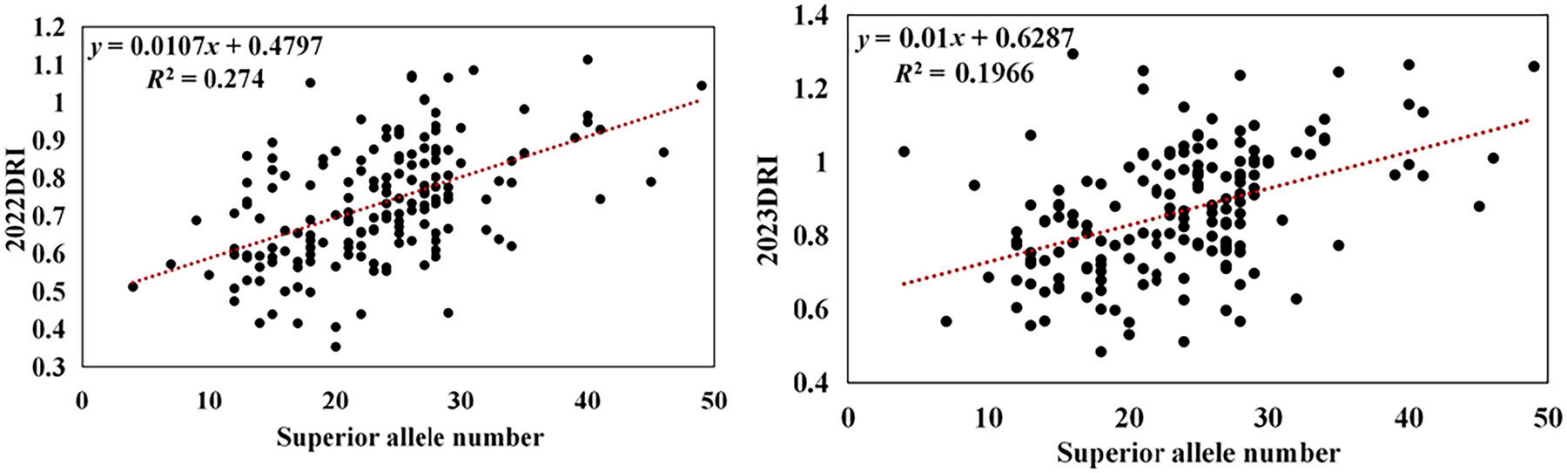
Curve fitting of superior alleles and the drought resistance index (DRI).

Evaluation of superior allele frequencies in the population revealed an average frequency of 46.49%, with values ranging from 5.71% to 85.14% (Table S2). Evaluation of cumulative effects revealed a positive association between DRI and the accumulation of superior alleles in both 2022DRI and 2023DRI. Inbred lines harboring nearly 50 superior alleles displayed DRI values up to 1.3 (Figure 4).

### 3.4 Identification of drought‐related candidate genes

**FIGURE 5:**
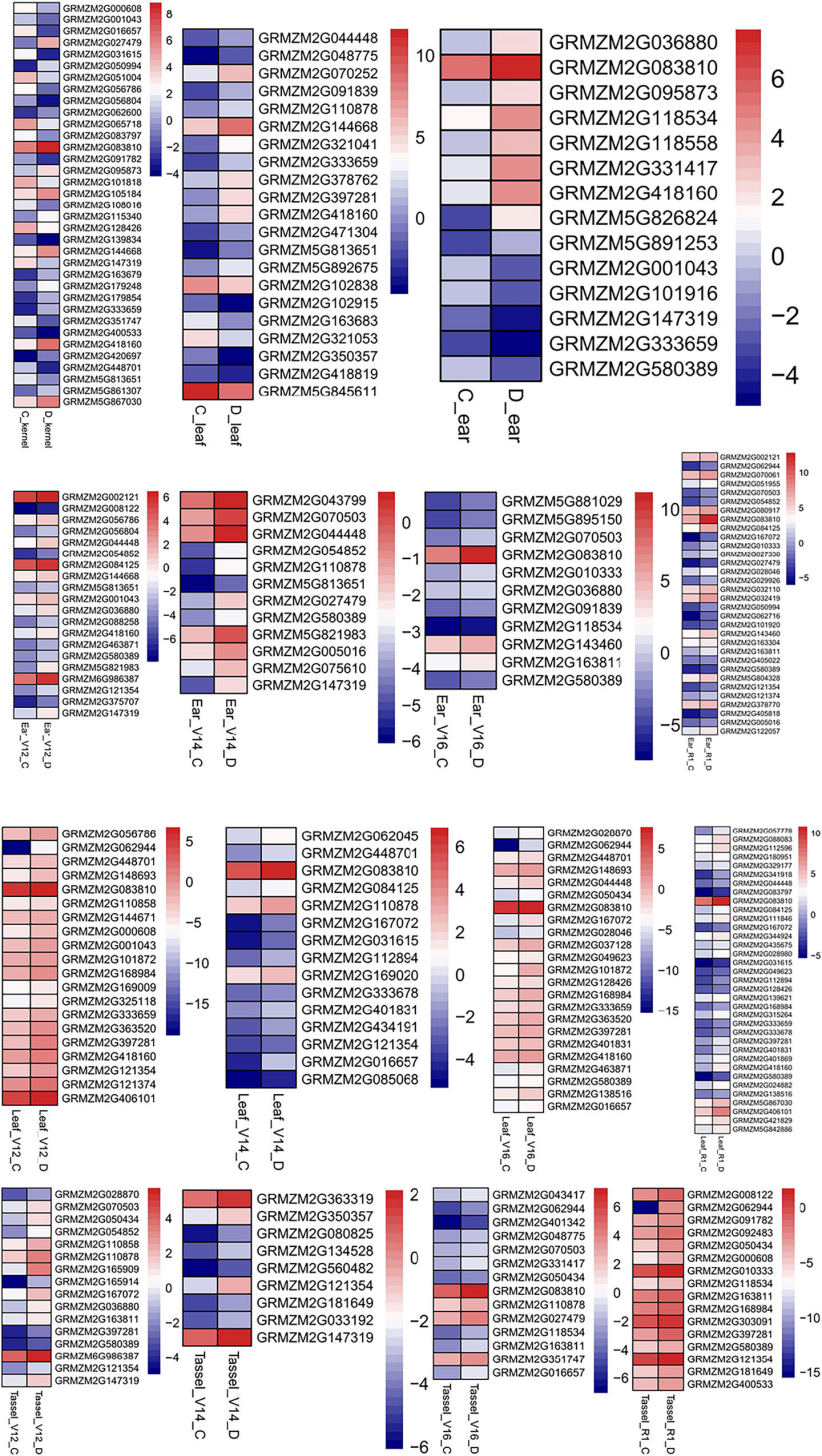
Expression profiles of candidate genes based on RNA‐seq data. (C and D) Control and drought treatments; V12, V14, and V16 correspond to the 12‐, 14‐, and 16‐leaf stages, respectively, while R1 represents the silking phase.

A total of 751 candidate genes were detected within 200‐kb regions flanking the 55 DRI‐associated SNPs, of which 91 genes were located near six reproducible loci. Combining two RNA‐seq datasets (GSE132113 and GSE71723), 60 and 118 genes were identified as differentially expressed under drought and normal conditions, respectively (Figure 5). Among them, 43 genes were shared between both datasets and were designated as core candidate genes (Table 2). Notably, several genes, including *GRMZM2G418160*, *GRMZM2G056804*, *GRMZM2G056786*, and *GRMZM2G397281*, were located near the six reproducible loci.

**TABLE 2:** Functional annotation of core candidate genes.

Gene ID	Chr	Start	End	Annotation
GRMZM5G813651	3	170066207	170069008	NAC (NAM, ATAF, and CUC) domain‐containing protein 35
GRMZM2G580389	4	236729924	236749742	—
GRMZM2G418160	4	202217985	202228510	Protein ROOT HAIR DEFECTIVE 3 homolog
GRMZM2G333659	4	15625718	15635892	Disease resistance protein RPM1
GRMZM2G147319	9	23019888	23022301	Zinc finger (C3HC4‐type RING finger) family protein
GRMZM2G144668	3	8980485	8982638	1‐Aminocyclopropane‐1‐carboxylate oxidase
GRMZM2G118534	4	16651299	16653987	–
GRMZM2G083810	3	8887555	8888747	17.5 kDa heat shock protein(class II)
GRMZM2G056804	1	10976281	10977368	RING‐H2 finger ATL16
GRMZM2G056786	1	10969158	10970735	RING‐H2 finger ATL16
GRMZM2G050994	4	236625023	236625831	—
GRMZM2G044448	3	229084600	229086448	Tetratricopeptide repeat (TPR)‐like superfamily protein
GRMZM2G036880	4	236369092	236370422	Chlorophyll a‐b binding protein 6A
GRMZM2G027479	4	204450542	204451323	—
GRMZM2G001043	4	14745233	14746187	—
GRMZM2G002121	1	1175065	1175912	GroES‐like zinc‐binding alcohol dehydrogenase family protein
GRMZM2G005016	8	163589594	163592085	—
GRMZM2G010333	4	236263370	236263969	—
GRMZM2G016657	8	157529051	157530072	Germin‐like protein
GRMZM2G031615	4	16440811	16449027	Auxin‐responsive protein
GRMZM2G049623	4	201966993	201971338	Pumilio homolog 12
GRMZM2G054852	3	103339567	103341320	Legumin‐like protein
GRMZM2G062944	1	113011443	113012754	—
GRMZM2G070503	2	236854869	236856413	—
GRMZM2G084125	3	8891773	8892776	Putative uncharacterized protein
GRMZM2G101872	4	237270999	237271985	Early nodulin‐like protein 9
GRMZM2G110878	3	228987815	228989858	—
GRMZM2G112894	4	20534915	20536583	Putative membrane lipoprotein
GRMZM2G121354	6	81777728	81781038	Proline‐rich spliceosome‐associated (PSP) family protein
GRMZM2G128426	4	204550921	204556706	Putative DUF869 domain containing family protein
GRMZM2G138516	5	87448785	87452289	Putative clathrin assembly protein
GRMZM2G143460	4	235451687	235454316	Heptahelical transmembrane protein 2
GRMZM2G148693	2	236669675	236679078	Agamous‐like MADS‐box protein AGL8
GRMZM2G163811	4	236148593	236149073	—
GRMZM2G167072	3	228965086	228968666	TPR_REGION domain‐containing protein
GRMZM2G168984	4	236937715	236939770	OSJNBa0058K23.8‐like protein
GRMZM2G333678	4	15638404	15640330	Tetratricopeptide‐like helical
GRMZM2G363520	4	235367805	235369289	Putative RING zinc finger domain superfamily protein
GRMZM2G397281	4	202281040	202282913	EGF(Epidermal Growth Factor)‐like domain‐containing protein
GRMZM2G401831	4	237194437	237217944	—
GRMZM2G406101	6	81864594	81872819	DNA‐binding protein RHL1
GRMZM2G448701	1	6148853	6149887	—
GRMZM5G821983	4	236149879	236150500	—

Abbreviation: Chr, chromosome.

**FIGURE 6:**
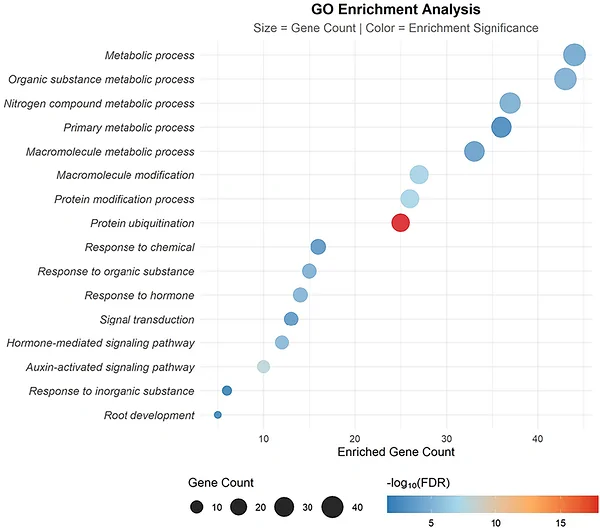
Gene Ontology (GO) enrichment analysis of the 43 candidate genes.

GO enrichment analysis revealed significant enrichment of 43 candidate genes in metabolism‐, signaling‐, and stress‐related biological processes (Figure 6). Metabolic processes included organic substance metabolic process and macromolecule metabolic process. Signaling‐related categories encompassed signal transduction, hormone‐mediated signaling pathway, and auxin‐activated signaling pathway. Stress response‐related terms comprised response to chemical, response to organic substance, response to inorganic substance, and root development. Protein ubiquitination displayed the strongest enrichment across all categories. Their functions have not been definitively confirmed experimentally, and their specific biological roles still require further experimental validation.

### 3.5 Analysis of protein interaction for candidate genes

**FIGURE 7:**
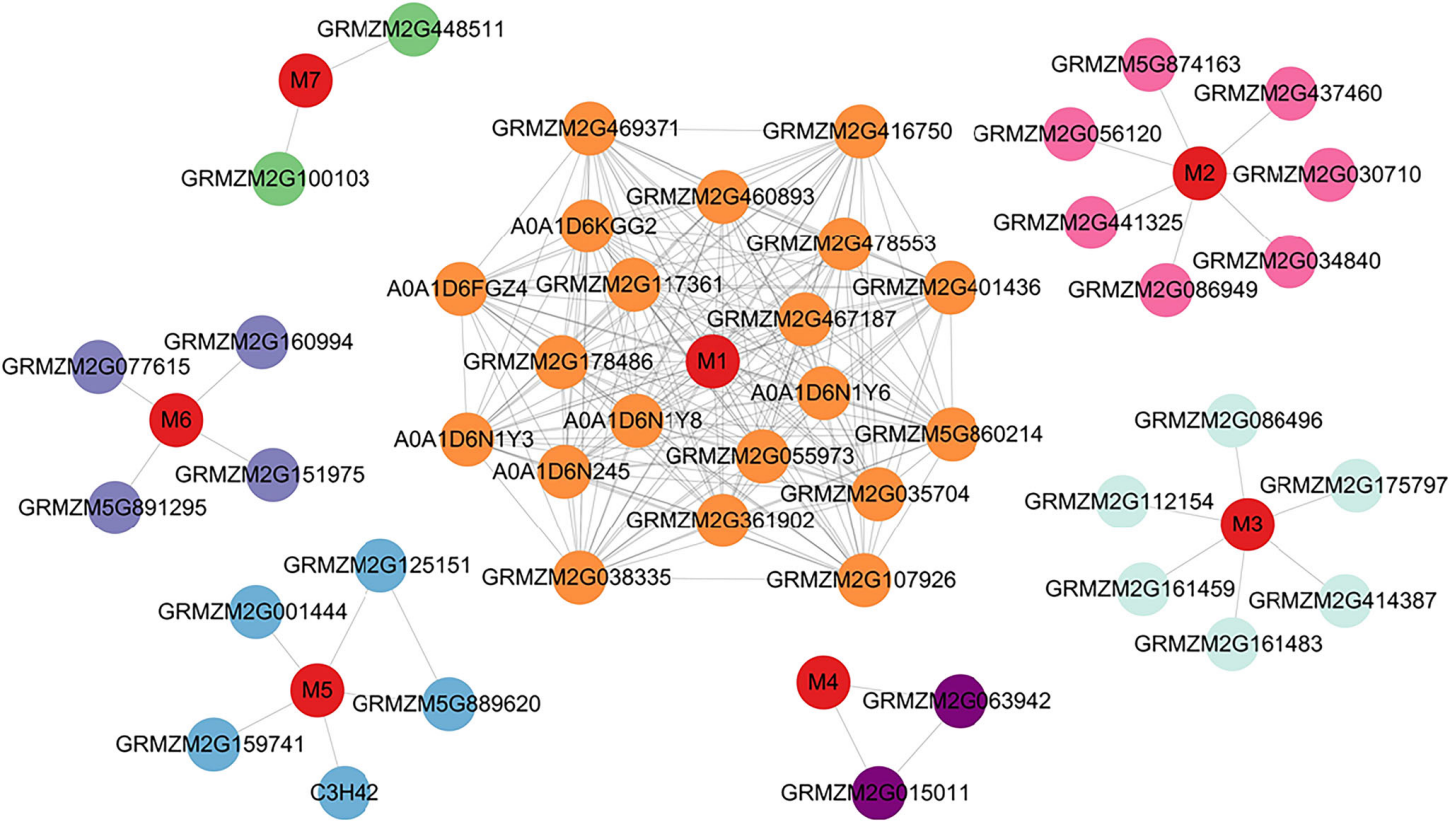
Protein–protein interaction (PPI) network of proteins encoded by candidate genes. Each node represents a protein, and edges denote predicted interactions. Proteins encoded by genes identified in this study are highlighted in red, whereas interacting proteins from different modules are distinguished by varying colors.

Analysis of protein–protein interaction demonstrated that seven candidate genes interacted with other functional proteins (Figure 7). For example, the M1 model (*GRMZM2G056786*) is annotated as a RING‐H2 finger protein ATL16, and *ZmATL10* overexpression was found to confer greater tolerance in response to heat (Ding et al., 2024). The M2 model (*GRMZM2G031615*) encodes an auxin‐responsive factor (ARF). Previous studies demonstrated that ARF proteins significantly enhance drought resilience in maize (L. Liu et al., 2024). The M3 model (*GRMZM2G144668*), an ACC oxidase that converts ACC to ethylene, participates in aerenchyma development in rice and maize (Z. Yang et al., 2023). The M4 model (*GRMZM2G406101*) encodes a nucleus‐localized protein that participates in the initiation of root hairs (Schneider et al., 1998). The M6 model (*GRMZM2G333678*), annotated as a TPR protein, is implicated in regulating plant growth and tolerance to abiotic stresses (X. Liu et al., 2024). The M7 model (*GRMZM2G083810*), annotated as a hsp17.6 protein, functions as a stress‐inducible molecular chaperone (Ma et al., 2021).

## 4 DISCUSSION

Drought is one of the major abiotic stresses limiting maize yield, and understanding its genetic basis is important for the development of drought‐tolerant varieties (B. Wang et al., 2019). In forward genetics, morphological, physiological, and biochemical responses to drought, such as plant height, transpiration rate, and chlorophyll content, provide useful indicators for evaluating germplasm tolerance (Anjum et al., 2016; Muhammad et al., 2023; Sun et al., 2021; C. Zhao et al., 2018). Previous GWAS on maize drought tolerance have mainly focused on indirect traits, particularly seedling stage and root‐related characteristics, including root system architecture, chlorophyll content, and survival rate (Jin et al., 2023; Moussa et al., 2021; D. Wang et al., 2025). Although these traits provide valuable insights into early drought responses, their direct relevance to final grain yield under field conditions is limited (Qu et al., 2025; Tollenaar & Lee, 2002). In contrast, the DRI, derived from grain yield evaluated under WW and WS conditions, provides a more direct and integrative measure of drought tolerance across the whole growth period and has clearer practical relevance for breeding (Du et al., 2025). However, few studies have genetically dissected maize drought tolerance using DRI as the primary trait.

GWAS for DRI and GY in the 200‐line panel identified 126 significant SNPs across 2022 and 2023, including loci associated with DRI, GY under WS and GY under WW conditions. The PVE of individual loci was generally moderate, ranging from 1.47% to 10.67% for DRI‐associated SNPs and from 4.64% to 10.67% for SNPs associated with GY under WS conditions. These moderate PVE values are not unexpected, as previous studies have shown that both drought tolerance and grain yield are complex quantitative traits in maize (R. Li et al., 2025; Y. Zhao & Su, 2019). Nineteen SNPs are detected under both water conditions, indicating partial overlap in the genetic control of GY under WW and WS conditions, consistent with previous studies (X. Li et al., 2017). However, most associated SNPs differed between WW and WS, suggesting QTL detection was strongly influenced by environmental conditions (R. Li et al., 2025; N. Wang et al., 2016). Only six SNPs showed stable associations with DRI across years, among which Affx‐291389821 and Affx‐88998430 were associated with multiple traits and environments, suggesting that these loci represent robust genetic signals for drought tolerance in maize. Among these six loci, four SNPs were selected for allelic effect analysis, and all four showed consistent favorable effects on DRI across years, indicating stable genetic effects across years and potentially limited genotype‐by‐environment interaction. Moreover, the positive relationship between DRI and the number of superior alleles supports an additive genetic basis for drought tolerance (H. Li et al., 2013; Tollenaar & Lee, 2010).

In addition, the significantly associated loci were enriched in several genomic bins, particularly 3.04, 4.08, 4.09, 5.04, and 10.02, suggesting that these regions may represent important genomic hotspots for drought‐related traits. Fourteen SNPs associated with yield or DRI are located in bin 3.04, which has previously been linked to root traits and GY‐related traits under WS conditions (Z. Liu et al., 2017; Messmer et al., 2009). Fifteen associated SNPs are located in bin 4.08, a known QTL hotspot for yield‐related traits (Nie et al., 2019). In bin 4.09, five DRI‐associated SNPs were detected in a region previously reported to harbor QTLs for drought‐related traits, including anthesis‐silking interval and ear height under water stress, as well as days to anthesis across environments (Messmer et al., 2009; X. Zhao et al., 2018). Another cluster region was bin 5.04, where seven SNPs were significantly associated with DRI. This bin has been reported as a pleiotropic region containing numerous QTLs for traits such as root structure, flowering time, plant morphology, and cell wall integrity (Barrière et al., 2015; Gu et al., 2017; Ren et al., 2019; Samayoa et al., 2014). Although these QTLs are not all directly linked to drought tolerance, the traits they regulate are closely associated with plant adaptation to drought stress.

In addition to genes near reproducible loci, several transcription factors were identified among the candidate genes. *GRMZM5G813651* (*NAC35*) belongs to the NAC transcription factor family, whose members are widely involved in plant growth regulation and stress adaptation (Xiong et al., 2025). Previous studies have shown that *ZmNAC55* functions as a drought‐response suppressor and that its suppression enhances drought tolerance by reducing water loss, improving survival, and decreasing membrane ion leakage (Fan et al., 2024). Furthermore, *GRMZM2G148693*, a member of the MADS‐box gene family, may participate in plant growth, development, and responses to abiotic stress (Castelán‐Muñoz et al., 2019; W. Zhao et al., 2021). Another candidate gene, *GRMZM2G147319*, encodes a C3HC4‐type RING finger protein that has been reported to be associated with tolerance to drought, salinity, temperature, and oxidative stress (Kim et al., 2019; Yuan et al., 2013).

Some limitations of this study should be acknowledged. The population size and marker density may have limited the power and resolution of GWAS, and the candidate genes identified here still require direct functional validation.

## 5 CONCLUSION

In this study, GY was evaluated under two contrasting water regimes across 2 consecutive years. Genome‐wide association analysis identified 126 significant SNPs using the FarmCPU model, with 43 SNPs linked to GY under WS conditions and 55 SNPs associated with DRI. Among these loci, six SNPs showed consistent associations with DRI across years, representing robust genetic signals for drought tolerance in maize. Finally, 43 genes involved in biological processes related to metabolic regulation, signal transduction, and environmental stress responses were defined as core candidate genes, including four genes located near these reproducible SNP loci. After further validation, the stable loci and candidate genes identified here may be useful for marker‐assisted selection and could also be integrated into genomic selection strategies to accelerate the breeding of drought‐tolerant maize varieties.

## AUTHOR CONTRIBUTIONS

**Haoyang Li**: Formal analysis; investigation; writing—original draft. **Xurong Hu**: Formal analysis. **Pengyan Zhang**: Formal analysis. **Yueying Zhao**: Formal analysis. **Yinglu Song**: Formal analysis. **Zheng Zhang**: Investigation. **Huahu Bu**: Investigation. **Jianzhong Chang**: Conceptualization; data curation; resources; supervision; writing—review and editing.

## CONFLICT OF INTEREST STATEMENT

The authors declare no conflicts of interest.

## Supporting information



Supporting Information

Supporting Information

## Data Availability

The genotypic datasets generated in this study have been deposited in the European Variation Archive (EVA; https://www.ebi.ac.uk/eva/) under the accession number PRJEB90981. Further inquiries can be directed to the corresponding author.
